# Geographic variation in cesarean delivery in the United States by payer

**DOI:** 10.1186/s12884-014-0387-x

**Published:** 2014-11-19

**Authors:** Rachel Mosher Henke, Lauren M Wier, William D Marder, Bernard S Friedman, Herbert S Wong

**Affiliations:** Truven Health Analytics, 150 Cambridge Park Drive, Cambridge, MA 02140 USA; U.S. Agency for Healthcare Research and Quality, 540 Gaither Road, Rockville, MD 20850 USA

**Keywords:** Cesarean delivery rate, Geographic variation, Medicaid, Private insurance

## Abstract

**Background:**

The rate of cesarean delivery in the United States is variable across geographic areas. The aims of this study are two-fold: (1) to determine whether the geographic variation in cesarean delivery rate is consistent for private insurance and Medicaid (2) to identify the patient, population, and market factors associated with cesarean rate and determine if these factors vary by payer.

**Methods:**

We used the Healthcare Cost and Utilization Project (HCUP) State Inpatient Databases (SID) to measure the cesarean rate at the Core-Based Statistical Area (CBSA) level. We linked the hospitalization data to data from other national sources to measure population and market characteristics. We calculated unadjusted and risk-adjusted CBSA cesarean rates by payer. For the second aim, we estimated a hierarchical logistical model with the hospitalization as the unit of analysis to determine the factors associated with cesarean delivery.

**Results:**

The average CBSA cesarean rate for women with private insurance was higher (18.9 percent) than for women with Medicaid (16.4 percent). The factors predicting cesarean rate were largely consistent across payers, with the following exceptions: women under age 18 had a greater likelihood of cesarean section if they had Medicaid but had a greater likelihood of vaginal birth if they had private insurance; Asian and Native American women with private insurance had a greater likelihood of cesarean section but Asian and Native American women with Medicaid had a greater likelihood of vaginal birth. The percent African American in the population predicted increased cesarean rates for private insurance only; the number of acute care beds per capita predicted increased cesarean rate for women with Medicaid but not women with private insurance. Further we found the number of obstetricians/gynecologists per capita predicted increased cesarean rate for women with private insurance only, and the number of midwives per capita predicted increased vaginal birth rate for women with private insurance only.

**Conclusions:**

Factors associated with geographic variation in cesarean delivery, a frequent and high-resource inpatient procedure, vary somewhat by payer. Using this information to identify areas for intervention is key to improving quality of care and reducing healthcare costs.

## Background

The rate of cesarean delivery in the United States increased about 60 percent from 1996 to 2009 and has since remained relatively stable, accounting for nearly one-third (32.7 percent) of all births in 2013 [1]. Increases for primary cesareans (i.e., those performed on women with no prior cesarean delivery) account for a large proportion of this overall growth [2]. Cesarean birth rates in the United States are higher than in other developed countries and are not associated with improved maternal or perinatal outcomes [3]. Indeed, cesarean delivery is associated with higher risk of maternal post-operative complications, including infection, blood transfusion, postoperative pain, and also increases the likelihood of future miscarriage, ectopic gestation, placenta previa, placenta accreta, and cesarean delivery [4]. Maternal stays for cesarean delivery tend to be longer and more costly than stays for vaginal delivery [5]. In light of evidence that lower rates of cesarean may reflect cost savings, higher quality of care, and reduced complications [6], an objective of Healthy People 2020 is to reduce cesarean births among women at low risk for vaginal birth complications [7].

Geographic variation in cesarean delivery has been documented [8-11], and variation in procedure rates may indicate underutilization or overutilization [12]. Specific factors associated with these differences have not been elucidated. Variation in cesarean delivery rates may be attributable to obstetric practice patterns [6,10,13], financial incentives [14,15], and legal concerns [16] rather than maternal risk profile or request [8,13]. One study using nationwide data from the Healthcare Cost and Utilization Project (HCUP) reported tenfold variation across hospitals in cesarean delivery rates [10]. The authors found that variation was not explained by hospital bed size, teaching status, geographic location, or clinical risk factors; rather, practice patterns are a likely driver of cesarean delivery variation. A study in England found that patient characteristics did not account for all of the variation in rates of cesarean delivery, and that rates of emergency cesarean appeared to be a key factor in the variation [11]. Another recent study found that hospital service area variation in obstetric practice patterns, malpractice climate and population-level characteristics explained little of the variation in the increase in cesarean delivery rates [17].

In 2010, Medicaid financed nearly half (48 percent) of all births in the United States [18]. The proportion of births covered by Medicaid varies considerably by state, with only one-quarter (24 percent) of births in Hawaii financed by Medicaid and more than two-thirds (69 percent) of births in Louisiana. Patients with private insurance have a higher rate of cesarean delivery compared to those who have Medicaid [5]. This suggests small area cesarean delivery rates and factors driving variation may be payer-specific. Area differences in payer compensation for birth, specifically, differences in the comparable generosity of reimbursement for cesarean section, may contribute to this variation. Payer-specific differences in provider response to clinical factors and access of care may also contribute.

Policymakers need a better understanding of the factors driving cesarean deliveries to inform efforts to decrease unnecessary medical care. In particular, knowledge about the role of nonclinical drivers of variation in cesarean delivery may provide valuable insight into potential mechanisms to reduce less medically appropriate interventions. If factors predicting cesarean deliveries for privately insured patients are different from factors predicting cesarean deliveries for Medicaid patients, interventions to reduce unnecessary care may need to be payer-specific. Little is known about the cause of differences in cesarean section between Medicaid and private insurance. This study will contribute to the literature and to the knowledge base by examining payer-specific differences in c-section variation and factors associated with variation.

Using hospital administrative data linked to other national data sources, we first examine variation in cesarean section rate at the CBSA level, overall and by payer. Next, we use data at the discharge level to examine patient, population, and market factors associated with geographic variation in primary cesarean rates in the United States overall and by payer. We hypothesize that factors associated with cesarean rate variation will differ by payer, given dissimilarities in the reimbursement of cesarean delivery and in access to care between Medicaid and private insurance.

## Methods

Data from the 2009 Healthcare Cost and Utilization Project (HCUP) State Inpatient Databases (SID) were used to examine variation in discharge rates for primary cesarean delivery by payer [19]. HCUP is a family of health care databases and related software tools and products developed through a federal-state-industry partnership and sponsored by the Agency for Healthcare Research and Quality (AHRQ). HCUP databases bring together the data collection efforts of state data organizations, hospital associations, private data organizations, and the federal government to create a national information resource of patient-level health care data. HCUP includes the largest collection of longitudinal hospital care data in the United States, with all-payer, encounter-level information beginning in 1988. The HCUP SID contain the universe of inpatient discharge abstracts from participating states, translated into a uniform format to facilitate multi-state and local market comparisons and analyses. All investigators signed a Data Use Agreement; because HCUP does not involve human subjects, IRB approval was not required for this study.

The following 44 states were included in the analysis: Arizona, Arkansas, California, Colorado, Connecticut, Florida, Georgia, Hawaii, Illinois, Indiana, Iowa, Kansas, Kentucky, Louisiana, Maine, Maryland, Massachusetts, Michigan, Minnesota, Missouri, Montana, Nebraska, Nevada, New Hampshire, New Jersey, New Mexico, New York, North Carolina, Ohio, Oklahoma, Oregon, Pennsylvania, Rhode Island, South Carolina, South Dakota, Tennessee, Texas, Utah, Vermont, Virginia, Washington, West Virginia, Wisconsin, and Wyoming. HCUP 2009 data was not available for the following 6 states: Alabama, Alaska, Delaware, Idaho, Mississippi and North Dakota.

We aggregated HCUP inpatient data from community, acute care hospitals to the Core-Based Statistical Area (CBSA) level using patient ZIP Code. CBSAs are the universe of U.S. metropolitan statistical areas and micropolitan areas [20] and are a readily available, transparent unit of analysis with established use in variation studies [21,22].

Our analytic file included 804 CBSAs (representing 86.5 percent of CBSAs in the United States). We obtained characteristics on population size, education, income, and race/ethnicity from the U.S. Census Bureau at the CBSA level. Data on physician and hospital resources, including the total number of primary care physicians, obstetric and gynecologic physicians, physician assistants, and midwives per capita were obtained from the Area Health Resource Files. Information on hospital type and beds per capita was obtained from the American Hospital Association. Average malpractice payment data were from the National Practitioner Data Bank.

We used the AHRQ Inpatient Quality Indicator (IQI) definition of primary cesarean rate to define the population studied. IQIs reflect procedures whose use varies significantly across hospitals or geographic areas and include measures of utilization of procedures for which there are questions of overuse, underuse, or misuse [23]. High rates of these indicators may suggest inappropriate or inefficient delivery of care.

*Primary cesarean delivery rate* was defined as the number of cesarean deliveries, identified by diagnosis-related group (DRG) (370–371), Medicare severity diagnosis-related group (MS-DRG) (765–766), and International Classification of Diseases, Ninth Revision, Clinical Modification (ICD-9-CM) procedure codes (740, 741, 742, 744, 7499) without a hysterectomy procedure code (7491) per 1,000 deliveries. *Deliveries* were defined by delivery DRG (370–375) and MS-DRG (765–768; 774–775), and excluded deliveries with the following: any diagnosis of abnormal presentation, preterm birth, fetal death, or multiple gestation diagnosis codes; any breech procedure codes; and previous cesarean delivery diagnosis in any diagnosis field.

*Payer* was based on the expected payer as indicated in the discharge record. *Medicaid* includes fee-for-service and managed care Medicaid patients. Patients covered by the State Children’s Health Insurance Program (SCHIP) may be included. Because most state data do not identify SCHIP patients specifically, it is not possible to present this information separately. *Private insurance* includes Blue Cross, commercial carriers, and private HMOs and preferred provider organizations (PPOs). One CBSA with a higher than expected percentage of births with Medicare as the primary expected payer was excluded.

First, we calculated unadjusted and risk-adjusted CBSA cesarean delivery rates by payer. Risk-adjusted rates were calculated as observed cesarean delivery rate divided by expected cesarean delivery rate, multiplied by the overall CBSA average cesarean delivery rate. The expected delivery rate was estimated using a hierarchical logistic model where the outcome was type of delivery (1 = cesarean; 0 = vaginal) and CBSA was included as the second level. Specifically, we used the SAS (SAS Institute, Inc; Cary, NC) GLIMMIX procedure that fits statistical models to data with correlations or nonconstant variability, where the outcome may not be normally distributed [24]. Because type of delivery was specified as a dichotomous outcome, we specified a logit link and binomial distribution.

In the model, we adjusted for maternal and neonatal characteristics associated with an increased risk of cesarean delivery. These factors included maternal age and race, primary expected payer (in the all-payer model), infant birth weight, and maternal conditions including maternal distress, placenta previa, hypertension, pre-eclampsia, pre-existing or gestational diabetes, herpes, HIV, and prior myomectomy. We included race in the risk adjustment model despite the absence of clinical evidence that race should affect cesarean rates because previous research has found wide variability in the rate of indications for primary cesarean section by race/ethnicity [25,26]. We included primary expected payer in the all-payer model because we expected rates to differ by payer and did not want these differences to confound the overall rate calculation. Finally, we included the set of maternal conditions in the risk adjustment model as these clinical factors may be considered medical indications for cesareans [16,27-32]. We look specifically at the influence of all of these factors (race, payer, maternal conditions) in the second part of our study. We calculated the correlation in cesarean delivery rate among Medicaid and private insurance using Pearson’s correlation weighted for population size.

Second, we measured the predictors of having a cesarean delivery for each hospitalization. We estimated models with all hospitalizations together (regardless of payer) and separately by the primary payer for the hospitalization. We included patient-level and CBSA-level predictors to evaluate factors associated with cesarean delivery. Variables included *patient-level measures* (detailed above), as well as *population measures* (e.g., race, income, education) and *market measures* (e.g., hospital market share, bed size, teaching status, provider density, average malpractice payments, and HMO enrollment). Continuous variables were centered at population means. We excluded 46 CBSAs for which complete patient, population, and market data were unavailable.

## Results

Table 1 provides overall CBSA summary statistics (mean, standard deviation, and coefficient of variation). Table 2 shows unadjusted and adjusted primary cesarean rates at the CBSA level overall and by payer. Adjusted rates did not vary substantially from unadjusted rates. The overall adjusted primary cesarean rate was 17.7 percent. Deliveries billed to private insurance had a higher primary cesarean rate (18.9 percent) relative to those billed to Medicaid (16.4 percent). As indicated by the coefficient of variation, there was more variation in cesarean rate for Medicaid than private insurance. Medicaid and private insurance adjusted primary cesarean rates were reasonably well correlated (ρ = 0.76).

**Table 1:** **Overall core-based statistical area summary statistics**

	**Mean**	**Standard deviation**	**Coefficient of variation**
**Total population (N)**	331,792	1,095,916	3.30
**Hospital births (N)**	4,301	14,344	3.33
**Patient race (%)**			
White	77.15	18.37	0.24
African American	8.15	10.89	1.34
Hispanic	10.00	14.91	1.49
Other	4.70	6.49	1.38
**Other population characteristics**			
Population aged 25 years or older with bachelor’s degree or higher (%)	21.24	7.85	0.37
Population below poverty level (%)	15.49	5.12	0.33
**Gini Index**	0.44	0.03	0.07
**Hospital market share (Herfindahl-Hirschman Index)**	69.65	31.34	0.45
**Market characteristics**			
Average malpractice payment ($)	$350,846	$117,612	0.34
Acute care beds: teaching hospital (%)	21.64	34.95	1.62
Acute care hospitals: bed size 0–99 (%)	63.46	39.11	0.62
For-profit hospitals (%)	19.30	28.50	1.48
Acute care beds per 1,000 capita	1.65	0.86	0.52
Obstetric beds per 1,000 capita	0.22	0.13	0.58
Neonatal intensive care unit beds per 1,000 capita	0.04	0.07	1.90
**Professional support**			
Primary care medical doctors per 100,000 capita	55.19	19.37	0.35
Physician assistants per 100,000 capita	22.61	15.86	0.70
Obstetric/gynecologic physicians per 100,000 capita	8.04	3.99	0.50
Midwives per 100,000 capita	1.97	2.62	1.33

**SOURCE** Author calculations of HCUP SID data linked with other national data sources.

**Table 2:** **Distribution of core-based statistical area primary cesarean delivery rate, overall and by payer**

	**All payer**			**Medicaid**			**Private insurance**		
	**Mean**	**SD**	**CoV**	**Mean**	**SD**	**CoV**	**Mean**	**SD**	**CoV**
Unadjusted	0.173	0.045	0.259	0.165	0.049	0.299	0.187	0.052	0.279
Risk-adjusted	0.177	0.044	0.250	0.164	0.047	0.297	0.189	0.049	0.268

SOURCE Author calculations of HCUP SID data linked with other national data sources. NOTES *Abbreviations*: *SD*, Standard deviation; *CoV*, Coefficient of variation.

### Geographic regions

**Figure 1:**
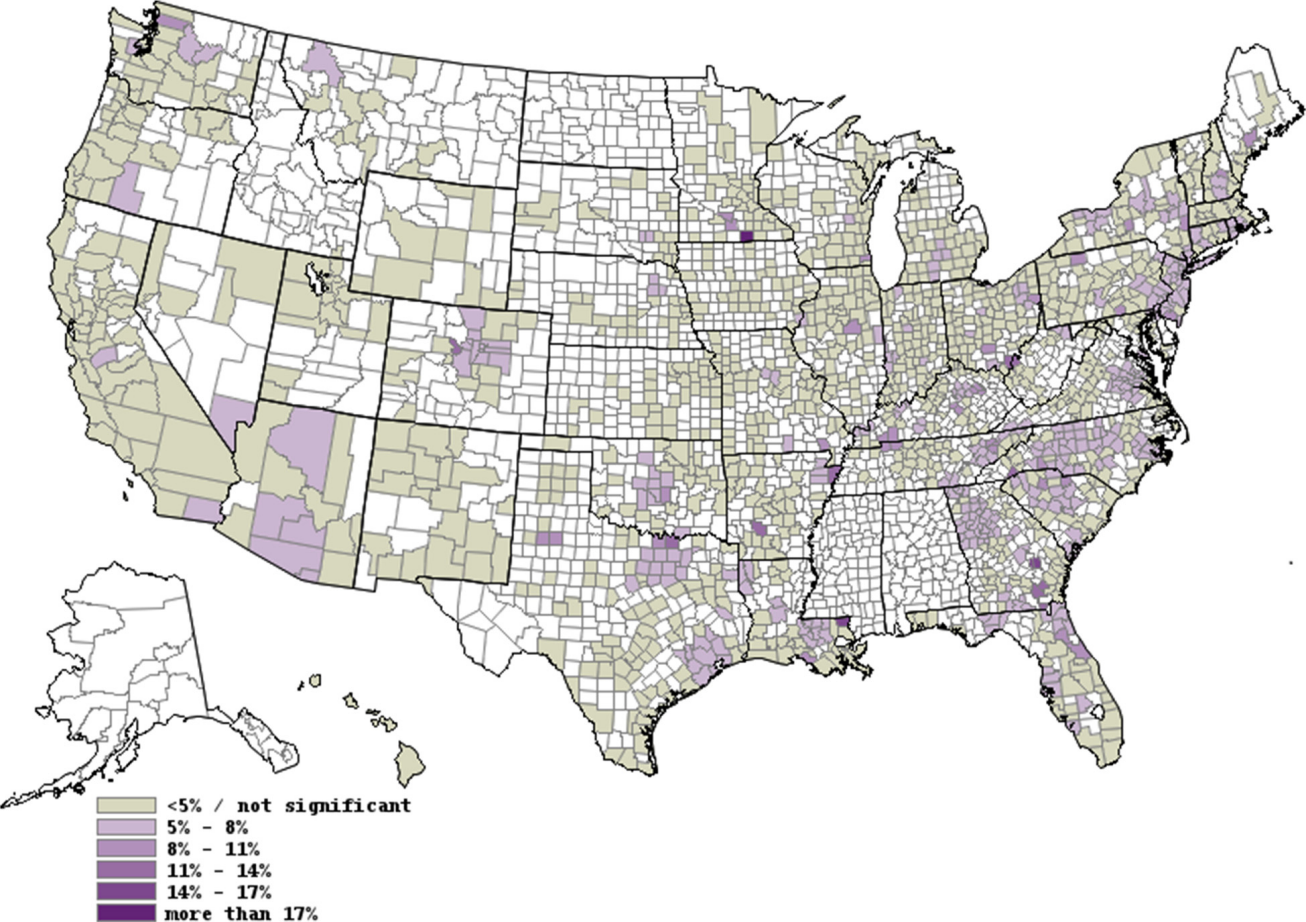
**Map displaying differences in private versus medicaid adjusted cesarean rates by core-based statistical area.** Unshaded areas represent areas that are not included in the analysis because they are rural or in states where hospital data are not available. County boundaries are shown.

Figure 1 displays significant differences in adjusted cesarean rates billed to private insurance versus Medicaid by CBSA. The purple shading indicates the estimate of the difference (Private – Medicaid).There appear to be clusters of CBSAs in some states (e.g., AZ, CT, FL, GA, OK, NC, SC, TX, VA, WA) where Medicaid cesarean rates are significantly lower than private insurance cesarean rates. There were no CBSAs that had private insurance cesarean rates that were significantly lower than Medicaid private insurance rates.

Table 3 shows the results of the hierarchical regression models predicting cesarean section for each hospitalization included in the sample. These models were estimated using discharge data from all payers and by payer (Medicaid, and private). Statistically significant estimates (*p* <0.05) are in bold. In general, discharge-level factors (e.g., patient measures) were stronger predictors of cesarean section then population and market characteristics.

**Table 3:** **Patient, population, and market factors associated with cesarean section by payer**

**Outcome = binary indicator of whether the birth was a cesarean section**	**All payer**			**Medicaid**			**Private**		
	**Births = 2,516,570**			**Births = 1,109,979**			**Births = 1,271,296**		
**Independent variables**	**Odds ratio**	**95% Confidence interval**		**Odds ratio**	**95% Confidence interval**		**Odds ratio**	**95% Confidence interval**	
**Age (years)**									
*Reference Group 18-24*									
Under 18	0.998	0.981	1.016	**1.046**	1.024	1.069	**0.875**	0.843	0.909
25–29	**0.891**	0.883	0.900	**0.794**	0.783	0.805	1.007	0.993	1.021
30–34	**0.876**	0.868	0.885	**0.821**	0.807	0.836	**0.954**	0.941	0.967
35–39	1.006	0.994	1.018	0.984	0.961	1.008	**1.079**	1.062	1.096
40–44	**1.317**	1.288	1.345	**1.236**	1.184	1.291	**1.438**	1.401	1.475
45–64	**2.085**	1.936	2.247	**1.650**	1.414	1.927	**2.392**	2.190	2.612
**Payer**									
*Reference Group Private*									
Medicaid	**0.773**	0.767	0.779						
Other	**0.707**	0.696	0.718						
**Patient race**									
*Reference Group White*									
Black	**1.113**	1.100	1.126	**1.062**	1.045	1.079	**1.176**	1.155	1.197
Hispanic	**0.850**	0.841	0.858	**0.780**	0.768	0.793	**0.965**	0.950	0.980
Asian	0.994	0.979	1.010	**0.908**	0.878	0.938	**1.045**	1.025	1.066
Native American	0.994	0.952	1.038	**0.875**	0.822	0.932	**1.124**	1.054	1.199
Other	**1.051**	1.033	1.070	0.974	0.948	1.001	**1.125**	1.099	1.152
Missing	0.997	0.980	1.015	**0.945**	0.915	0.975	**1.026**	1.002	1.051
**Infant birth weight**									
*Reference Group Normal*									
Very low (<1500 g)	**1.127**	1.038	1.224	1.114	1.002	1.238	**1.126**	1.009	1.257
Low (1500 < 2500 g)	**1.964**	1.375	2.806	**1.937**	1.145	3.275	**1.822**	1.088	3.051
High (4500 g+)	1.074	0.877	1.314	1.374	0.956	1.975	0.899	0.697	1.160
**Maternal conditions**									
Maternal distress, not specified	**1.970**	1.610	2.411	**2.018**	1.510	2.697	**1.870**	1.396	2.504
Placenta previa	**14.75**	14.04	15.49	**14.673**	13.49	15.963	**14.776**	13.850	15.763
Hypertension	**2.081**	2.053	2.110	**2.128**	2.082	2.174	**2.042**	2.006	2.078
Pre-eclampsia	**1.679**	1.646	1.712	**1.631**	1.583	1.679	**1.723**	1.680	1.767
Diabetes	**1.840**	1.819	1.862	**1.968**	1.930	2.007	**1.772**	1.744	1.800
Herpes	**3.062**	2.950	3.178	**3.819**	3.608	4.042	**2.552**	2.426	2.686
HIV	**2.986**	2.729	3.268	**3.428**	3.078	3.818	**2.201**	1.834	2.641
Prior myomectomy	**22.60**	20.73	24.64	**17.41**	14.80	20.49	**24.85**	22.36	27.63
**Population characteristics**									
Percentage of population 25 years or older with bachelor’s degree or higher	**0.993**	0.989	0.997	**0.994**	0.990	0.998	**0.994**	0.990	0.998
Percentage of population below poverty level	0.998	0.992	1.004	0.995	0.987	1.003	0.998	0.992	1.004
African American – Percentage in the population	**1.003**	1.001	1.005	1.001	0.999	1.003	**1.005**	1.003	1.007
Hispanic – Percentage in the population	1.001	0.999	1.003	1.001	0.999	1.003	1.001	0.999	1.003
Other – Percentage in the population	0.998	0.994	1.002	0.998	0.994	1.002	0.998	0.994	1.002
Gini Index	**1.027**	1.015	1.040	**1.023**	1.011	1.035	**1.033**	1.020	1.045
**Market characteristics**									
Hospital market share 1–100 (Herfindahl-Hirschman Index)	**0.999**	0.999	0.999	**0.999**	0.997	1.001	**0.998**	0.998	0.998
Average malpractice payment	1.000	1.000	1.000	1.000	1.000	1.000	1.000	1.000	1.000
Number of hospital births	1.001	0.999	1.003	1.001	0.999	1.003	1.001	0.999	1.003
Percentage of acute care beds: teaching hospital	1.000	1.000	1.000	1.000	1.000	1.000	1.000	1.000	1.000
Percentage of acute care hospitals: bed size 0–99	1.000	1.000	1.000	1.000	1.000	1.000	1.000	1.000	1.000
Percentage of for-profit hospitals	1.000	1.000	1.000	1.000	1.000	1.000	1.000	1.000	1.000
Acute care beds per 1,000 capita	1.051	1.011	1.093	1.067	1.022	1.114	1.031	0.990	1.075
OB beds per 1,000 capita	0.936	0.744	1.177	0.923	0.710	1.200	1.028	0.807	1.311
Neonatal ICU beds per 1,000 capita	0.959	0.906	1.015	0.964	0.905	1.026	0.950	0.900	1.004
Primary care medical doctors per 100,000 capita	**0.997**	0.995	0.999	**0.997**	0.995	0.999	**0.997**	0.995	0.999
OB/GYN physicians per 100,000 capita	**1.012**	1.004	1.020	1.007	0.999	1.015	**1.014**	1.006	1.022
Midwives per 100,000 capita	**0.991**	0.983	0.999	0.993	0.983	1.003	**0.991**	0.983	0.999

SOURCE Author calculations of HCUP SID data linked with other data sources.

NOTES Model estimates are from 2 level hierarchical linear models with discharges specified as the first level and CBSAs specified as the second level. Boldface represents statistically significant results (*p* < .05). Abbreviations: *ICU*, Intensive care unit; *GYN*, Gynecologic; *OB*, Obstetric.

### Patient measures

Across all three models, older age was associated with increased odds of cesarean delivery. One exception was the youngest age group (mothers under 18) was associated with a higher odds of cesarean compared to the 19–24 age group for patients with Medicaid. Interestingly, the youngest group had lower odds of cesarean in the private insurance model. For all payers, maternal Black race was a positive predictor of cesarean, and Hispanic was a negative predictor of cesarean. Asian and Native American backgrounds were associated with a decreased likelihood of cesarean for stays billed to Medicaid but an increased likelihood for stays billed to private insurance.

Of the clinical characteristics, we found that very low infant birth weight (<1500 g) was a positive predictor of cesarean delivery compared to normal weight infants in the overall model and for private insurance. Low birth weight (1500 < 2500 g) was also a positive predictor of cesarean delivery, irrespective of payer. Maternal conditions such as maternal distress, placenta previa, hypertension, pre-eclampsia, pre-existing or gestational diabetes, herpes, HIV, and prior myomectomy were consistently positive and strong predictors of cesarean delivery, regardless of payer. However, the magnitude of the association for herpes and HIV was notably larger for Medicaid, and the magnitude of the association for prior myomectomy was notably higher for private insurance.

### Population measures

Higher population educational attainment was a predictor of lower cesarean delivery for both payers, but income was not significant. For the average CBSA, a one percentage change in the Gini Index—meaning greater income inequality—was also associated with an increase in the odds of a cesarean section across all models. The percentage of African Americans in the population was associated with an increased likelihood of cesarean delivery overall and for private insurance but not for Medicaid.

### Market measures

Several of the market measures we examined had an impact on cesarean delivery. A 10 unit higher Herfindahl-Hirschman Index—indicating a more consolidated hospital market—was associated with a one percent decreased likelihood of cesarean, irrespective of payer. Acute care beds per capita were significant positive predictors of cesarean delivery in the all-payer and Medicaid models but not the private insurance model.

The concentration of OB/GYN and primary care physicians per capita were also predictors of cesarean delivery. A ten-unit increase in primary care physicians per capita was associated with a three percent decrease in odds of cesarean delivery, regardless of payer. For all payers and stays billed to private insurance, a ten-unit increase in OB/GYN physicians per capita was associated with a 12 percent increase in odds of cesarean delivery in the all payer model and a 14 percent increase in odds of cesarean delivery in the private insurance model; this relationship was not significant for Medicaid. In contrast, a ten-unit increase in midwives per capita was associated with a three percent decrease in the odds of cesarean delivery for all payers and stays billed to private insurance. This relationship was not significant for Medicaid. Ideally, we would have used midwife indicator as a patient-level variable but this data was not available for the vast majority of the states. We did, however, conduct a sensitivity analysis that included an indicator of whether a midwife was the hospital provider in a model limited to the states where this information was available. This analysis found that having a hospital provider that was a midwife was statistically significantly associated with a vaginal birth.

We found no relationship between the following market characteristics and cesarean section: average malpractice payments, number of hospital births, percentage of teaching hospitals, percentage of small hospitals, percentage of for-profit hospitals, OB beds per 1,000 capita, neonatal ICU beds per 1,000 capita.

## Discussion

This study demonstrates geographic variations at the CBSA level in primary cesarean delivery rate, even after adjusting for observable maternal and neonatal characteristics that are associated with increased likelihood of cesarean delivery. The minimal difference between the unadjusted and adjusted CBSA cesarean delivery rate suggests that variation is not largely attributable to differences in patient mix.

Our finding that the average adjusted CBSA cesarean delivery rate for women with Medicaid is lower than the CBSA cesarean delivery rate for women with private insurance suggests potential overuse of this service. Our analysis at the hospitalization level that found Medicaid is an independent predictor of vaginal delivery is consistent with studies conducted with older data [25], and adds to the evidence that payer type influences provider decision-making regarding type of delivery (vaginal vs cesarean section).

In a novel analysis, we found that patterns of variation in cesarean section at the CBSA-level were somewhat different for Medicaid compared to private insurance. Although there was a reasonably high correlation between Medicaid and private insurance cesarean rates, there was more variation in cesarean delivery for Medicaid compared to private insurance at the CBSA level. These differences in rates by payer can be partially explained by the differing relationships between population and market characteristics and cesarean delivery by payer. For example, the concentration of midwives and OB/GYN physicians per capita each had a significant influence on private insurance cesarean section rates but not on Medicaid rates. The number of acute care beds per capita had a significant influence on Medicaid caesarean section rates but not on private insurance rates. Additionally, although we could not examine the influence of reimbursement for cesarean section on the rates, it is possible that the generosity of cesarean section reimbursement influences cesarean rates and thus contributes to the payer specific variation observed [14].

An unexpected finding was that women with Asian and Native American backgrounds were more likely to have a cesarean section if they were privately insured and were less likely to have a cesarean section if they had Medicaid. Previous research has found an association between race/ethnicity and cesarean section [26], but this is the first finding of a differential association by payer. More research is needed to understand why this may be the case.

Previous research indicates that having an obstetrician as the primary medical provider is associated with increased likelihood of cesarean delivery [32], whereas patients receiving care from midwives have fewer labor abnormalities and a lower incidence of cesarean delivery [33]. Indeed, we found that for stays billed to all payers and to private insurance, OB/GYN physicians per capita were a positive predictor for cesarean delivery. Midwives per capita were associated with reduced likelihood of cesarean for all payers and for stays billed to private insurance. It was unexpected that midwife supply was unrelated to cesarean rate for Medicaid, but this could be because of unequal access to midwifery practices by payer.

Primary care physicians per capita in the CBSA were associated with a reduction in cesarean delivery, irrespective of payer. The number of primary care physicians per capita may reflect improved access to primary care services and earlier access to prenatal services, which may reduce complications during pregnancy and, ultimately, the need for cesarean deliveries. This study provides evidence of a link between primary care supply and reduced propensity for cesarean section. Thus, it reinforces the importance efforts to bolster primary care practice underway as part of the Affordable Care Act [34].

We found that areas with higher levels of income inequality had higher cesarean delivery rates, irrespective of payer. This is consistent with research that has found individuals in areas with greater income inequality report poorer health, regardless of insurance type [35]. Worse overall health may reflect reduced access to preventative care or other area factors resulting in health differences. Additional research is needed to better understand the implications of this finding.

Our analysis has several limitations. First, we relied on administrative data that provide detail on utilization of inpatient services but do not capture information on non-hospital births, such as birthing centers or home births, which may result in underestimating the effect. Second, data were not available on unobserved patient characteristics (e.g., number of previous births) and other characteristics (e.g., type of provider who delivered baby, maternal body mass index, prenatal care, hospital obstetric care guidelines and policies) that may predispose patients to receive a cesarean. We did not have clinical details on reasons for cesarean delivery. If we had more complete information on medical indications for cesarean section and if the distribution of the missing medical indications on cesarean section varied by payer, we would expect a wider difference between the Medicaid and private insurance CBSA risk-adjusted cesarean rate than was observed. We also did not have data on maternal preference for cesarean deliveries, which may account for a portion of cesarean deliveries [36]. Third, reimbursement data were not readily available, so we were not able to examine the impact of reimbursement differential for cesarean versus vaginal delivery on cesarean outcome.

There are several important strengths of using HCUP data to study geographic variation in cesarean delivery, including national reach, comprehensiveness of inpatient information and payer detail, and ability to link to other data sources for additional information on population and market characteristics.

## Conclusion

In this study, we found significant variation in the rate of cesarean section at the CBSA level even after adjusting for patient mix. When medically appropriate, cesarean delivery represents an important intervention for improving maternal and neonatal outcomes. However, cesarean deliveries are a costly intervention and are associated with myriad complications, including higher risk of maternal readmission for surgical site and uterine infection [37]. Mothers who undergo cesarean sections often deliver via repeat cesarean for future births which, in turn, further drives increases in cesarean rates. Importantly, this potentially unwarranted geographic variation in medical care may be an indicator of poor quality of care.

Understanding the driving forces of geographic variation in frequent and high-resource inpatient procedures, such as cesarean delivery, is key to improving quality of care and reducing healthcare costs. The present study found evidence that area cesarean section rates may be driven by population and market characteristics such as educational attainment, area inequality, hospital competition, hospital capacity, and physician supply. We also found that variation in rates differs for Medicaid compared to private insurance. To identify how these findings could be applied to reduce unnecessary variation, additional research would be helpful understand the underlying mechanism that leads these factors to influence cesarean rates. For example, future studies may survey or interview physicians, hospitals, patients, and other stakeholders involved in maternity care to probe why these population and market characteristics influence decision-making. To understand why certain patient characteristics have a differential impact on delivery decisions by payer, researchers could survey physicians using case vignettes of patients with various backgrounds that prompt for both a likely delivery decision (recommend cesarean or vaginal birth) and explanations behind that decision. Research in this area could lead to the development of clinical or policy interventions that attempt to reduce unnecessary variation in cesarean section rates across geographic areas.

## Abbreviations

AHRQ: Agency for healthcare research and quality
CBSA: Core-based statistical area
HCUP: Healthcare cost and utilization project
IQI: Inpatient quality indicator
SID: State inpatient databases
